# Large-scale plasma proteomics improves prediction of heart failure among MASLD individuals: A prospective cohort study

**DOI:** 10.1016/j.ajpc.2026.101662

**Published:** 2026-05-05

**Authors:** Zhi-Yuan Xiong, Si-Qi Chen, Hong-Xuan Huang, Shu-Min Lai, Ling Kuang, Hao-Jie Chen, Bing-Yun Zhang, Yi-Xin Wang, Ya-Xuan Li, Hui Zhu, Yan-Song Li, Er-La Huang, Dan Liu, Chen Mao, Zhi-Hao Li

**Affiliations:** Department of Epidemiology, School of Public Health, Southern Medical University, Guangzhou, 510515, Guangdong, China

**Keywords:** Plasma proteomics, Heart failure, Metabolic dysfunction-associated steatotic liver disease, Biomarkers, Risk prediction, Prospective cohort

## Abstract

**Background:**

Metabolic dysfunction-associated steatotic liver disease (MASLD) substantially elevates the risk of heart failure (HF). While large-scale proteomics improves HF prediction in general populations, its incremental predictive value beyond standard clinical models in MASLD remains unexplored.

**Objective:**

To identify plasma protein biomarkers for incident HF in MASLD and evaluate the predictive utility of integrating these signatures with the predicting risk of cardiovascular disease events (PREVENT) clinical model.

**Methods:**

We prospectively analyzed 17,091 individuals with MASLD at baseline. Multivariable and LASSO—Cox regressions were applied to 2911 plasma proteins to identify optimal predictors. Predictive discrimination and reclassification were assessed using Harrell’s C-index, time-dependent area under the curve (AUC), net reclassification improvement (NRI), integrated discrimination improvement (IDI), and decision curve analysis (DCA).

**Results:**

Over a median follow-up of 13.56 years, 953 incident HF events occurred. Integrating the PREVENT model with a 37-protein panel substantially improved predictive discrimination (C-index 0.805 vs. 0.723; ΔC-index 0.082, 95%CI: 0.064–0.100). Moreover, a parsimonious model containing only 5 proteins (NT-proBNP, WFDC2, LTBP2, BCAN, HAVCR1) delivered a meaningful incremental improvement over the PREVENT baseline (C-index 0.769 vs. 0.723; ΔC-index 0.046, 95%CI: 0.027–0.064). Pathway analyses indicated these proteins associating with systemic inflammation and extracellular matrix remodeling.

**Conclusions:**

Large-scale proteomics significantly enhances HF risk prediction in MASLD, providing a robust tool for identifying high-risk individuals who may benefit from intensive clinical monitoring and preventive strategies.

## Graphical abstract

**Figure:**
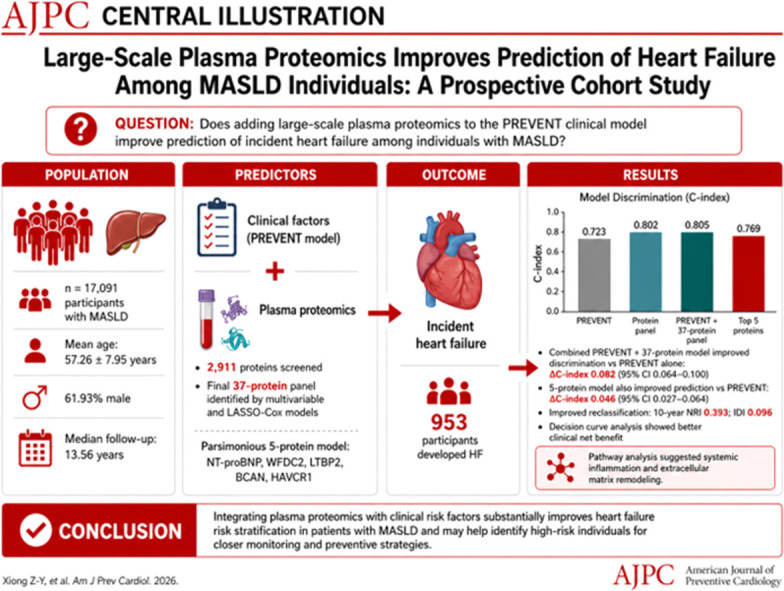

## 1 Introduction

Metabolic dysfunction-associated steatotic liver disease (MASLD), defined by hepatic steatosis with at least one cardiometabolic risk factor, is increasingly recognized as a major contributor to cardiovascular morbidity and mortality [1]. Heart failure (HF) is a leading cause of cardiovascular mortality, affecting over 64 million people worldwide and imposing a substantial healthcare burden [2,3]. Emerging evidence indicates that MASLD substantially elevates the risk of HF [4,5], underscoring the need for improved risk stratification and precision prevention in this vulnerable population [6].

Large-scale circulating proteomics provide significant potential for identifying clinically actionable biomarkers and improving risk quantification beyond what is captured by conventional epidemiological risk factors [7,8]. Previous proteomic studies have identified several protein biomarkers associated with incident HF in general population, offering insight into the proteomic signatures associated with cardiovascular risk [[9], [10], [11]]. For instance, NT-proBNP is a well-established marker of myocardial stretch and cardiac stress and a robust predictor of incident HF [[12], [13], [14]]. The potential of proteomic profiling lies in its ability to unveil novel biomarkers that may precede the onset of HF [15]. Most proteomic HF studies have focused on general populations; to our knowledge, no large-scale proteomic investigation has specifically characterized HF risk among individuals with MASLD.

Conventional risk calculators for cardiovascular primary prevention, such as Pooled Cohort Equation [16], Systematic COronary Risk Evaluation 2 (SCORE2) [17], QResearch Cardiovascular Risk Score 3 (QRISK3) [18], were derived primarily to predict cardiovascular disease. These equations generally incorporate traditional risk factors without consideration of risk enhancing factors on HF, such as chronic inflammatory diseases [18,19]. The American Heart Association recently introduced the Predicting Risk of Cardiovascular disease Events (PREVENT) equations for absolute risk assessment of total cardiovascular disease, including HF [20]. Recently, the PREVENT equations have been evaluated and shown clinical utility for cardiovascular risk stratification in MASLD populations [21]. However, the potential incremental value of proteomic biomarkers for enhancing PREVENT prediction in MASLD remains unexplored.

We hypothesize that integrating the PREVENT model with a large-scale proteomic panel may substantially refine HF risk stratification in MASLD. Our objectives were to: (i) identify plasma proteins associated with incident HF, and (ii) evaluate whether incorporating proteomic biomarkers improve risk prediction beyond the validated PREVENT framework.

## 2 Methods

### 2.1 Study population

The UK Biobank (UKB) is a population-based, prospective cohort study that enrolled >500,000 adults between 2006 and 2010 [22]. Baseline assessments included demographic, psychosocial, and medical questionnaires; standardized physical measurements; and biospecimen collection [23]. Detailed information regarding the study design and participant has been published previously [22]. The UKB received approval from the North West Multicentre Research Ethics Committee, Manchester (REC reference number: 11/NW/0382), and all participants provided written informed consent.

Among 52,533 participants with plasma proteomics, we excluded those without MASLD (n = 32,437). We further excluded individuals with baseline pre-existing liver diseases (n = 1290) [24], identified via primary care, hospital admissions data using International Classification of Diseases-10 (ICD-10) codes K70-K77 (alcoholic or toxic liver disease, hepatic failure, chronic hepatitis, fibrosis and cirrhosis, and other inflammatory liver diseases). We then excluded those with diagnosed HF (ICD-10 codes; I11.0, I13.0, I13.2, I50.0, I50.1, I50.9) at baseline (n = 301), and participants with self-reported HF, angina, myocardial infarction, or pulmonary oedema at baseline (n = 1364). Participants who withdrew consent or were lost to follow-up (n = 50) were also excluded, yielding a final analytic cohort of 17,091 (Supplementary Figure 1). For internal validation employing a 3:1 split, the cohort was randomly split into a training set (n = 12,819) for primary analyses and a test set (n = 4272) for validation.

### 2.2 Ascertainment of MASLD

MASLD was defined as the presence of hepatic steatosis accompanied by one or more cardiometabolic criteria [25]: (i) body mass index (BMI)  ≥  25 kg/m^2^ or waist circumference (WC) >  94 cm for males or 80 cm for females; (ii) fasting plasma glucose (FBG) ≥ 100 mg/dL or type 2 diabetes or treatment for type 2 diabetes; (iii) blood pressure ≥  130/85 mmHg or specific antihypertensive drug treatment; (iv) triglycerides (TG) ≥  150 mg/dL or lipid‐lowering medication; and (v) high‐density lipoprotein (HDL) cholesterol <  40 mg/dL for males and <  50 mg/dL for females or lipid‐lowering medication. We used the fatty liver index (FLI) as the primary indicator for assessing steatosis. Individuals with FLI ≥ 60 were classified as having steatosis, consistent with prior study [26].

$$FLI=\;\frac{e^{0.953\times \text{ln}\left(\text{TG}\right)+0.139\times \text{BMI}+0.718\times \text{In}\left(\text{GGT}\right)+0.053\times \text{WC}-15.745}}{1+e^{0.953\times \text{ln}\left(\text{TG}\right)+0.139\times \text{BMI}+0.718\times \text{In}\left(\text{GGT}\right)+0.053\times \text{WC}-15.745}}\times 100$$

### 2.3 Ascertainment of outcome

The outcome was defined as the incident HF during the follow-up period after an MASLD diagnosis. Incident HF events after recruitment were identified via linkage to primary care, hospital admissions, and death registers [27]. Records with the ICD-10 codes (I11.0, I13.0, I13.2, I50.0, I50.1, I50.9) were considered HF cases. The follow-up was calculated from the date of study enrollment until the diagnosis of HF, death, or the end of follow-up (^October 31, 2022^), whichever occurred first.

### 2.4 Proteomic measurements

Baseline plasma samples from > 50,000 participants were quantified via the Olink Explore platform using a proximity extension assay (PEA) for high-throughput proteomic analysis. Detailed protocols for sample handling, plasma processing, data analysis, and quality control have been previously described [28]. A total of 2923 proteins were quantified and expressed as normalized protein expression (NPX) values. NPX values were standardized to Z-scores prior to analysis. Proteins with > 20% missing data (n = 12) were excluded, leaving 2911 proteins for analysis. Values below the limit of detection (LOD) were set to the LOD [29], and remaining missing values (≤ 20%) were imputed using protein-specific means to preserve individual protein distributions in high-dimensional data. This processing workflow followed established methods from prior literature [30].

### 2.5 Assessment of covariates

Covariates were selected based on the PREVENT model [20] and included: age, sex (male, female), systolic blood pressure (SBP, mmHg), BMI (kg/m^2^), smoking status (never, previous, current), antihypertensive medication use (yes, no), history of diabetes (yes, no), Townsend deprivation index (TDI, ranging from 0 to 1), urine albumin-to-creatinine ratio (UACR, mg/g), glycated hemoglobin (HbA1c, mmol/mol), and estimated glomerular filtration rate (eGFR, mL/min/1.73 m^2^).

The SBP was measured by the Omron 705 IT electronic blood pressure monitor. BMI was calculated by dividing weight (kg) by the square of height (m^2^) [31]. TDI examines the material well-being of individuals and households, with a higher score indicating greater deprivation [32]. The UACR is a simple method for assessing urinary albumin excretion, commonly used to detect early kidney damage and an important biomarker for cardiovascular disease risk [33]. The eGFR is an independent predictor of cardiovascular disease, estimated using the Chronic Kidney Disease Epidemiology Collaboration formula [34]. Antihypertensive medications use and history of diabetes were defined as self-reported or physician-diagnosed; diabetes was additionally defined by HbA1c >6.5%.

### 2.6 Statistical analysis

Baseline characteristics are presented as numbers (percentages) for categorical variables and means (standard deviations [SD]) for continuous variables, stratified by incident HF status. Group differences were assessed using the *χ*^2^ test (categorical variables) and the two-sample *t-*test (continuous variables), as appropriate. To increase the statistical power, we used multiple imputations by chained equations to assign any missing values of covariates [35]. The distribution of missing values across covariates is provided in Supplementary Table 1.

For predictive modeling, the cohort was randomly partitioned into a training set (2/3; n = 12,819) and a validation set (1/3; n = 4272) (Supplementary Figure 1). In the training set, multivariable Cox proportional hazards regression was first utilized to evaluate the association between individual proteins (per 1-SD increase in NPX) and incident HF. Specifically, clinical covariates (age, sex, TDI, BMI, smoking status, SBP, eGFR, UACR, HbA1c, antihypertensive medication use, and history of diabetes) were fixed as baseline adjustments. Each of the 2911 proteins was then sequentially incorporated into the model to estimate hazard ratios (HRs) and 95% confidence intervals (CIs). Statistical significance for this initial screening was defined using a rigorous Bonferroni-adjusted threshold of *P* < 0.05/2911. The proportional hazards assumption was verified using Schoenfeld residuals, with no violations detected [36].

To further select the most robust predictors, all proteins that passed the initial Bonferroni-adjusted screening were simultaneously entered into a Cox model with a least absolute shrinkage and selection operator (LASSO) penalty. The PREVENT clinical covariates were kept unpenalized while the candidate proteins were subjected to penalization. The penalty parameter (λ) was determined via 10-fold cross-validation based on Harrell's C-index. To enforce model parsimony and strictly prevent overfitting, we selected the λ value located within one standard error (SE) of the optimal performance metric to identify the predictive protein panel (Supplementary Figure 2).

For HF prediction, three models were evaluated in the validation set: (i) PREVENT model: age, sex, TDI, BMI, smoking status, SBP, eGFR, UACR, HbA1c, antihypertensive medication use, and diabetes history; (ii) protein panel: only integrating proteins selected by LASSO; and (iii) combined model: PREVENT covariates plus the LASSO-selected proteins. Model performance was assessed in the validation set using Harrell’s C-index, time-dependent area under the curve (AUC), continuous/categorical net reclassification improvement (NRI) and integrated discrimination improvement (IDI). Decision curve analysis (DCA) was performed to calculate and compare the net benefit of these models across a range of clinically relevant risk thresholds, against the strategies of intervening in all or no patients [37].

To ensure the robustness of our findings we conducted several sensitivity analyses. First, to validate the identified protein-HF associations, we reconstructed Cox models after excluding incident HF cases occurring within the first 2 years of follow-up to mitigate reverse causality and excluding participants with extreme protein values (outside mean ± 3 SDs). Proteins maintaining *P* < 0.05 across these conditions were considered robust. Second, Fine-Gray competing risk and cumulative incidence function were used to account for the competing risk of non-HF mortality [38]. Third, model performance was reassessed in a subgroup where MASLD was strictly defined by the magnetic resonance imaging–proton density fat fraction (MRI-PDFF) reference standard (≥ 5.0%) [39]. Fourth, sequential forward selection evaluated the incremental prognostic value of tiered protein subsets based on LASSO coefficients to derive parsimonious predictive model.

To gain mechanistic insights, pathway enrichment analyses for the LASSO-selected proteins were conducted using Gene Ontology (GO) [40], Kyoto Encyclopedia of Genes and Genomes (KEGG) [41], and Reactome databases [42]. To rigorously account for multiple testing, pathways were considered significantly enriched only if they met a Bonferroni-adjusted threshold of *P* < 0.05/N, where N represents the total number of pathways identified in the enrichment analysis.

All statistical analyses were performed via R version 4.4.1. Two-sided *P*-value were reported, and *P* < 0.05 with Bonferroni correction was considered statistically significant [43].

## 3 Results

### 3.1 Characteristics of participants

A total of 17,091 individuals with MASLD were included (mean age: 57.26 ± 7.95 years; 61.93% male). Participants who developed HF were older, more likely to be male and current or former smokers, and had higher TDI, BMI, systolic blood pressure and HbA1c levels compared to those without HF (all *P* < 0.05; Table 1). Baseline characteristics presented similar distribution between the training set (n = 12,819) and validation set (n = 4272) (Supplementary Table 2).

**Table 1:** Baseline characteristics of the participants.

**Characteristics**	**Overall**		**Incident HF**			***P* value**
	(n = 17,091)		No (n = 16,138)		Yes (n = 953)	
**Age**	57.26 (7.95)		56.99 (7.94)		61.80 (6.49)	<0.001
**Sex**						0.052
Female	6506 (38.07)		6172 (38.25)		334 (35.05)	
Male	10,585 (61.93)		9966 (61.75)		619 (64.95)	
**Smoking status**						<0.001
Never	8525 (49.88)		8149 (50.50)		376 (39.45)	
Previous	6668 (39.01)		6235 (38.64)		433 (45.44)	
Current	1898 (11.11)		1754 (10.87)		144 (15.11)	
**TDI**	−0.98 (3.25)		−1.01 (3.24)		−0.55 (3.33)	<0.001
**BMI, kg/m^2^**	31.43 (4.47)		31.35 (4.42)		32.74 (5.07)	<0.001
**WC, cm**	102.28 (9.88)		102.04 (9.74)		106.42 (11.26)	<0.001
**SBP, mmHg**	142.65 (17.43)		142.46 (17.33)		146.00 (18.75)	<0.001
**eGFR, mL/min/1.73m^2^**	93.33 (13.97)		93.64 (13.74)		88.00 (16.60)	<0.001
**UACR, mg/g**	23.27 (46.18)		22.35 (44.22)		38.85 (69.78)	<0.001
**GGT, U/L**	54.10 (55.16)		53.71 (54.35)		60.58 (67.15)	0.002
**HDL, mmol/L**	1.26 (0.30)		1.26 (0.30)		1.25 (0.34)	0.120
**TG, mmol/L**	2.39 (1.20)		2.39 (1.20)		2.30 (1.16)	0.015
**HbA1c, mmol/mol**	37.81 (8.55)		37.59 (8.19)		41.51 (12.70)	<0.001
**Antihypertensive medication**						<0.001
No	12,036 (70.42)		11,566 (71.67)		470 (49.32)	
Yes	5055 (29.58)		4572 (28.33)		483 (50.68)	
**Diabetes**						<0.001
No	15,916 (93.13)		15,119(93.69)		797 (83.63)	
Yes	1175 (6.87)		1019 (6.31)		156 (16.37)	

Continuous variables are expressed as mean (SD), and categorical variables are described as number (percentage). Abbreviations: HF, heart failure; BMI, body mass index; WC, waist circumference; SBP, systolic blood pressure; TDI, Townsend deprivation index; eGFR, estimated glomerular filtration rate; UACR, urine albumin-to-creatinine ratio; GGT, gamma-glutamyl transferase; HDL, high-density lipoprotein; TG, triglycerides; HbA1c, glycated hemoglobin.

### 3.2 Associations between plasma proteins and incident HF

**Fig. 1:**
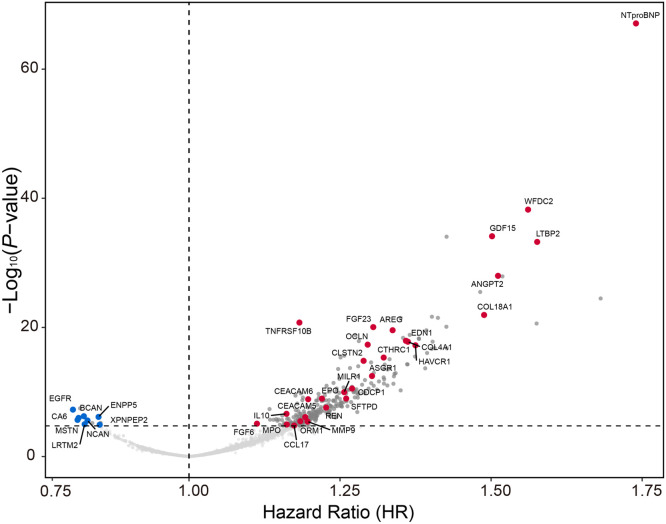
Associations between plasma proteins and risk of HF in MASLD. HRs represent hazard ratios per 1-SD increase in normalized protein expression (NPX), Models were adjusted for age (years, continuous), sex (female, male), TDI (continuous), BMI (kg/m^2^, continuous), smoking status (never, previous, current), SBP (mmHg, continuous), eGFR (mL/min/1.73 m^2^, continuous), UACR (mg/g, continuous), HbA1c (mmol/mol, continuous), antihypertensive medication (yes, no), history of diabetes (yes, no). The proteins lying above the dotted line were *P* value <0.05/2911 (Bonferroni-correction). Red/blue dots were proteins further selected by LASSO regression in the training set (n = 12,819).

During a median (interquartile range) follow-up of 13.56 (12.76‒14.35) years, 953 participants developed HF. Multivariable Cox regression analyses identified 301 of the 2911 proteins significantly associated with incident HF. LASSO Cox regression in the training set identified a parsimonious panel of 37 proteins that optimized predictive performance (Fig. 1, Supplementary Figure 2 and Supplementary Table 3). According to the LASSO coefficients, the most important positive associations were observed for NT-proBNP (HR = 1.74; 95%CI: 1.63‒1.85), followed by WFDC2 (HR 1.56; 95%CI:1.46–1.67) and LTBP2 (HR 1.58; 95%CI:1.46–1.70). The strongest protective associations were observed for BCAN (HR 0.83; 95%CI:0.77–0.89), NCAN (HR 0.83; 95%CI:0.77–0.90), and MSTN (HR 0.82; 95%CI:0.75–0.89) (Table 2).

**Table 2:** Leading proteins associated with HF in MASLD individuals.

**Protein**	**Gene**	**Panel**	**HR (95%CI)**^1^	***P* value**
**Top five proteins positively associated with HF risk**				
N-terminal pro-B-type natriuretic peptide	NT-proBNP	Cardiometabolic	1.74 (1.63–1.85)	8.43×10^−68^
WAP four-disulfide core domain protein 2	WFDC2	Oncology	1.56 (1.46–1.67)	5.74×10^−39^
Latent-transforming growth factor beta-binding protein 2	LTBP2	Cardiometabolic	1.58 (1.46–1.70)	5.71×10^−34^
Hepatitis A virus cellular receptor 1	HAVCR1	Oncology	1.37 (1.28–1.48)	5.91×10^−18^
Tumor necrosis factor receptor superfamily member 10B	TNFRSF10B	Neurology	1.18 (1.14–1.22)	5.19×10^−18^
**Top five proteins inversely associated with HF risk**				
Brevican core protein	BCAN	Neurology	0.83 (0.77–0.89)	5.70×10^−7^
Neurocan core protein	NCAN	Neurology	0.83 (0.77–0.90)	2.59×10^−6^
Growth/differentiation factor 8	MSTN	Cardiometabolic	0.82 (0.75–0.89)	2.10×10^−6^
Leucine-rich repeat and transmembrane domain-containing protein 2	LRTM2	Neurology_II	0.83 (0.76–0.90)	9.79×10^−6^
Xaa-Pro aminopeptidase 2	XPNPEP2	Oncology	0.85 (0.79–0.92)	1.17×10^−5^

^2^*P* values were adjusted by Bonferroni correction. Abbreviations: HF, heart failure; MASLD, metabolic dysfunction-associated steatotic liver disease; HR, hazard ratio.

1 Models were adjusted for age (years, continuous), sex (female, male), TDI (continuous), BMI (kg/m^2^, continuous), smoking status (never, previous, current), SBP (mmHg, continuous), eGFR (mL/min/1.73 m^2^, continuous), UACR (mg/g, continuous), HbA1c (mmol/mol, continuous), antihypertensive medication (yes, no), history of diabetes (yes, no).

### 3.3 Development and primary performance of predictive models

**Fig. 2:**
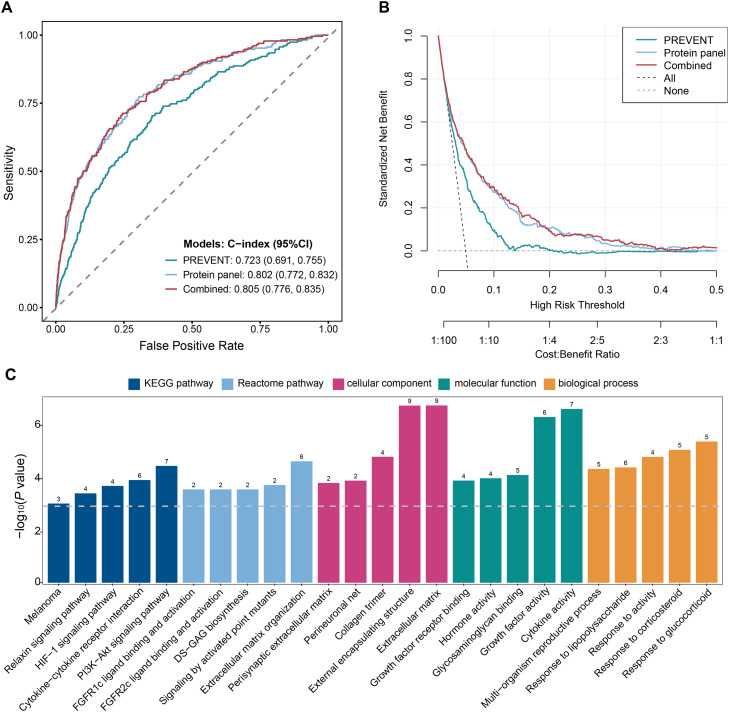
Predictive performance and functional pathway analysis of the proteomic models for incident HF in MASLD. A Receiver operating characteristic (ROC) curves for the PREVENT, Protein panel, and Combined model in the MASLD cohort. B Decision curve analysis (DCA) evaluating the clinical utility of the three models at a median follow-up of 13.56 years. The net benefit of each model is assessed against the reference strategies of intervening in all or no patients. C Functional enrichment analysis of proteins associated with HF pathogenesis. The horizontal dashed line denotes the Bonferroni corrected statistical significance threshold (*P* < 0.05/184; where 184 represents the total enriched pathways counts); all entries above the line are defined as significantly enriched. The number labeled on top of each bar indicates the count of proteins enriched in the corresponding pathway/functional term.

In the validation set, the PREVENT model yielded a Harrell’s C-index of 0.723 (95%CI: 0.691–0.755). The protein panel comprising the 37 LASSO-selected proteins achieved a C-index of 0.802 (95%CI: 0.772–0.832), while the combined model reached 0.805 (95%CI: 0.776–0.835), and ΔC-index of 0.082 (95%CI: 0.064–0.100) (Fig. 2A). Time-dependent AUC analyses confirmed this predictive superiority across multiple follow-up horizons. The combined model demonstrated 5-, 10-, and 15-year AUCs of 0.822, 0.815, and 0.846, respectively, compared with 0.652, 0.711, and 0.761 for the PREVENT model (Table 3).

**Table 3:** Model predictive value in validation set (n = 4272).

**Characteristics**	**PREVENT model**	**Protein panel**	**Combined model**
**Harrell’s C-index**			
C-index (95%CI)	0.723 (0.691–0.755)	0.802 (0.772–0.832)	0.805 (0.776–0.835)
C-index increment (95%CI)	Reference	0.078 (0.060–0.096)	0.082 (0.064–0.100)
**Time-dependent AUC**			
5-year AUC (95%CI)	0.652 (0.580–0.723)	0.835 (0.775–0.895)	0.822 (0.758–0.885)
5-year AUC increment (95%CI)	Reference	0.183 (0.119–0.231)	0.170 (0.112–0.217)
10-year AUC (95%CI)	0.711 (0.668–0.754)	0.820 (0.781–0.859)	0.815 (0.776–0.855)
10-year AUC increment (95%CI)	Reference	0.110 (0.066–0.153)	0.105 (0.069–0.143)
15-year AUC (95%CI)	0.761 (0.702–0.819)	0.841 (0.802–0.881)	0.846 (0.806–0.885)
15-year AUC increment (95%CI)	Reference	0.081 (0.032–0.149)	0.085 (0.039–0.146)
**Continuous NRI**			
5-year continuous NRI (95%CI)	Reference	0.461 (0.339–0.583)	0.470 (0.350–0.612)
10-year continuous NRI (95%CI)	Reference	0.315 (0.243–0.446)	0.393 (0.310–0.481)
15-year continuous NRI (95%CI)	Reference	0.288 (0.000–0.482)	0.321 (0.000–0.567)
**IDI**			
5-year IDI (95%CI)	Reference	0.073 (0.042–0.202)	0.074 (0.042–0.222)
10-year IDI (95%CI)	Reference	0.090 (0.067–0.163)	0.096 (0.076–0.184)
15-year IDI (95%CI)	Reference	0.123 (−0.025–0.251)	0.138 (−0.091–0.365)

Variables in the PREVENT model including age (years, continuous), sex (female, male), TDI (continuous), BMI (kg/m^2^, continuous), smoking status (never, previous, current), SBP (mmHg, continuous), eGFR (mL/min/1.73 m^2^, continuous), UACR (mg/g, continuous), HbA1c (mmol/mol, continuous), antihypertensive medication (yes, no), history of diabetes (yes, no). The protein panel was developed by integrating 37 LASSO-selected proteins, while the Combined model (Protein+PREVENT) incorporated 37 LASSO-selected proteins alongside the PREVENT variables. Abbreviations: PREVENT: Predicting Risk of Cardiovascular Disease Events; AUC, area under curve; NRI, net reclassification improvement; IDI, integrated discrimination improvement.

Risk reclassification metrics further underscored the added value of the proteomic panel. For 10-year HF risk prediction, the combined model improved continuous risk reclassification (continuous NRI: 0.393; 95%CI: 0.310–0.481) and overall integrated discrimination (IDI: 0.096; 95%CI: 0.076–0.184) relative to the PREVENT model. This indicates that the protein panel led to a net 39.3% improvement in overall risk ranking for heart failure prediction, and widened the absolute predicted risk gap between actual HF cases and non-cases by 9.6%.

DCA also demonstrated that the combined model consistently provided superior net clinical benefit across a broad range of clinically relevant risk thresholds (Fig. 2B). For instance, evaluated at a representative 10% risk threshold, a widely recognized clinical cut-off for initiating cardiovascular preventive pharmacotherapy [44], utilizing the combined model instead of the baseline PREVENT model yielded a net benefit equivalent to identifying 3.3 additional true HF cases per 1000 patients without increasing the number of false-positive interventions.

### 3.4 Sensitivity analyses

**Fig. 3:**
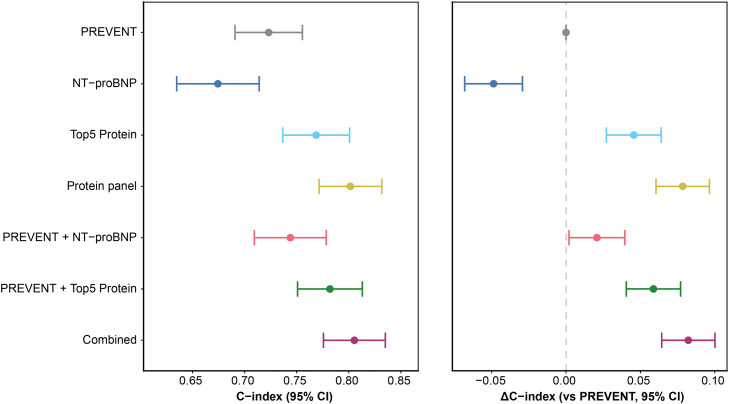
Harrell’s C-index and incremental discrimination of protein-based risk prediction models in the validation set. Left panel shows the C-index (with 95%CIs) for the baseline PREVENT model, top 5 proteins [NT-proBNP, WFDC2, LTBP2, BCAN, HAVCR1], protein panel, and combined model. Right panel shows the ΔC-index vs. the PREVENT model (with 95%CIs). Error bars represent 95%CIs. Abbreviations: CI, confidence interval; PREVENT, predicting risk of cardiovascular disease events.

To assess the robustness of our findings and the stability of the predictive models, we conducted a series of comprehensive sensitivity analyses. First, the identified protein-HF associations remained stable after applying stringent exclusions, including the removal of HF cases occurring within the first 2 years of follow-up and participants with extreme protein values (outside mean ± 3 SDs) (Supplementary Tables 3 and 4). Second, when accounting for competing risks using the Fine-Gray method, although non-HF mortality exceeded incident HF probability over the full follow-up (Supplementary Figure 3A), the combined model maintained higher discrimination for 10-year HF prediction than the PREVENT baseline (competing-risk adjusted AUC: 0.785, 95%CI: 0.744–0.826 vs. 0.707 95%CI: 0.664–0.749) (Supplementary Figure 3B). Third, in the MRI-PDFF sub-cohort (n = 1481; 29 incident HF events; Supplementary Table 5), the combined model increased the C-index from 0.818 (95%CI: 0.660–0.977) to 0.976 (95%CI: 0.940–1.000), with corresponding improvements in the full-follow-up AUC (from 0.820 to 0.979) and reclassification metrics (continuous NRI: 0.902; IDI: 0.414) (Supplementary Tables 6). Lastly, evaluation of tiered protein subsets showed that adding NT-proBNP to the PREVENT model (C-index: 0.723; 95%CI: 0.691–0.755) yielded a C-index of 0.744 (ΔC-index: 0.021, 95%CI: 0.002–0.040). Adding the top 5-protein (NT-proBNP, WFDC2, LTBP2, BCAN, HAVCR1) to PREVENT further and substantially improved discrimination (C-index:0.782; ΔC-index: 0.059, 95%CI: 0.041–0.077) and capturing a substantial proportion of the gain achieved by the full 37-protein panel (ΔC-index: 0.082, 95%CI: 0.064–0.100) (Fig. 3 and Supplementary Tables 7).

### 3.5 Pathway enrichment analysis

Pathway enrichment analyses revealed that HF-associated proteins were predominantly enriched in inflammatory and structural remodeling pathways. The most significantly enriched pathways in KEGG were PI3K-Akt signaling pathway (*P* = 4.17×10^−5^); GO enrichment indicates Cytokine activity (*P* = 3.06×10^−7^), Response to glucocorticoid (*P* = 5.11×10^−6^) and Extracellular matrix (ECM) (*P* = 2.26×10^−7^) in MF, BP and CC ontology; Reactome also indicated enriched ECM organization (*P* = 2.82×10^−5^) (Fig. 2C and Supplementary Table 8).

## 4 Discussion

In this prospective cohort of individuals with MASLD, we used high-throughput proteomics to identify biomarkers for HF risk prediction. Moreover, we identified 37 HF-associated proteins and developed a combined model that improved risk discrimination beyond the PREVENT clinical baseline. A parsimonious 5-protein model (NT-proBNP, WFDC2, LTBP2, BCAN, HAVCR1) provided comparable incremental predictive value to the full 37-protein panel. These findings offer a targeted, data-driven approach for refined HF risk stratification in the MASLD population.

Recent evidence indicates that standard cardiovascular risk assessment tools, such as the Framingham Risk Score and the PREVENT equations, exhibit suboptimal discrimination for cardiovascular events in patients with MASLD [21]. Consistent with this observation, our baseline PREVENT model yielded a C-index of 0.723, which is lower than the 0.79 to 0.85 typically reported in general populations [45]. This discrepancy suggests that traditional calculators, which rely on generic metabolic and hemodynamic indicators, may not fully account for the pathophysiological characteristics of MASLD [46]. MASLD contributes to incident HF through non-atherosclerotic mechanisms, including systemic inflammation, ectopic fat deposition, and profibrotic pathways, which can lead to early myocardial remodeling and diastolic dysfunction prior to overt cardiovascular events [47,48]. Our study identifies a proteomic signature associated with these biological alterations. The 37-protein panel improved discrimination compared to the PREVENT baseline (C-index: 0.805 vs. 0.723), and the 5-protein model retained predictive utility (C-index: 0.769), providing a targeted approach for HF risk prediction in MASLD.

The predictive value of the 5-protein panel aligns with the pathophysiology connecting MASLD to HF. The inclusion of NT-proBNP, a marker of myocardial stretch, is expected; however, our data suggest it is insufficient as a standalone predictor in this cohort. The additional prognostic value is derived from complementary markers, specifically WFDC2, LTBP2, BCAN, and HAVCR1, which are associated with ECM remodeling, cytokine activity, and fibrogenesis [49,50]. Specifically, WFDC2 and LTBP2 are implicated in both hepatic stellate cell activation and subsequent myocardial stiffening, forming a potential liver-specific molecular link to HF [51,52]. Furthermore, the inclusion of HAVCR1 (Kidney Injury Molecule-1) reflects subclinical renal tubular injury and microvascular inflammation, which are potent accelerators of volume overload and HF progression in metabolic phenotypes [53,54]. Concurrently, BCAN, a chondroitin sulfate proteoglycan, signifies aberrant systemic matrix turnover and neuro-hormonal stress associated with advanced metabolic dysfunction [55,56]. By reflecting multi-organ involvement, the 5-protein signature not only identifies MASLD-related cardiac injury beyond standard HF biomarkers but also offers increased flexibility for routine clinical assays.

### 4.1 Strengths and limitations

Our study has several strengths. First, the large-scale, prospective design with over a decade of follow-up provides adequate statistical power. Second, we applied conservative statistical approaches to mitigate the risk of overfitting in high-dimensional omics data. Third, distilling the proteomic data into a 5-protein panel provides a concise tool for potential clinical translation.

However, several limitations should be considered. First, reliance on the FLI for MASLD ascertainment may introduce misclassification bias. However, the robustness of our findings was confirmed in a rigorously defined MRI-PDFF sub-cohort, mitigating concerns regarding diagnostic misclassification. Second, the primary outcome was overall incident HF ascertained via electronic health records. The limited sample size of concurrent echocardiographic measurements, precluded adequately powered analyses stratified by HF subtype. Third, the limited availability of independent cohorts with concurrent MASLD phenotyping, high-throughput proteomics, and long-term HF surveillance constrained external validation. Although we maximized internal reliability through sensitivity analyses, future studies remain necessary to definitively establish the model's generalizability. Finally, the observational design precludes causal inference. While this proteomic panel offers substantial prognostic value, orthogonal biological confirmation is required to elucidate its potential mechanistic role.

## 5 Conclusions

This study identifies a robust circulating proteomic signature that significantly improves incident HF prediction in individuals with MASLD. Our study suggests the proteomic signature offers a pragmatic tool for identifying high-risk individuals who may benefit from intensive clinical monitoring and targeted preventive strategies.

## Author agreement statement

1. All authors have participated sufficiently in the work to take public responsibility for appropriate portions of the content, and have approved the final version of the manuscript for submission.
2. The manuscript is original, has not been published previously, and is not under consideration for publication elsewhere in any form or language.
3. All authors have disclosed any potential conflicts of interest (as detailed in the accompanying Declaration of Interest Statement), and no undisclosed conflicts exist.
4. The study complies with all ethical standards and relevant institutional review board requirements, and informed consent was obtained where applicable.
5. All authors agree to be bound by the journal’s editorial policies and the terms of the Journal Publishing Agreement that will be executed upon acceptance of the manuscript.

## Ethics approval and consent to participate

This study was conducted using the UK Biobank resource in accordance with the Declaration of Helsinki. The use of data from the UK Biobank was approved by the North West Multicentre Research Ethics Committee (11/NW/0382). This research was conducted under application number 806,778. All participants provided written informed consent.

## Consent for publication

Not applicable.

## Funding

This work was supported by the National Natural Science Foundation of China (grant number 82-204-115), the Guangdong Basic and Applied Basic Research Foundation (grant number 2025A1515010406).

## Data availability statements

The proteomic data used in this study are available from the UK Biobank upon reasonable request (https://www.ukbiobank.ac.uk/). The code used in this study is available from the corresponding author upon reasonable request.

## CRediT authorship contribution statement

**Zhi-Yuan Xiong:** Writing – original draft, Visualization, Formal analysis, Data curation. **Si-Qi Chen:** Writing – original draft, Visualization, Formal analysis, Data curation. **Hong-Xuan Huang:** Validation, Software. **Shu-Min Lai:** Validation, Software. **Ling Kuang:** Writing – review & editing, Supervision. **Hao-Jie Chen:** Methodology, Investigation. **Bing-Yun Zhang:** Methodology, Investigation. **Yi-Xin Wang:** Supervision. **Ya-Xuan Li:** Supervision. **Hui Zhu:** Supervision. **Yan-Song Li:** Supervision. **Er-La Huang:** Supervision. **Dan Liu:** Writing – review & editing, Supervision. **Chen Mao:** Writing – review & editing, Supervision. **Zhi-Hao Li:** Writing – review & editing, Project administration, Funding acquisition, Conceptualization.

## Declaration of competing interest

The authors declare the following financial interests/personal relationships which may be considered as potential competing interests:

Zhi-Hao Li reports financial support was provided by National Natural Science Foundation of China. Zhi-Hao Li reports financial support was provided by Guangdong Basic and Applied Basic Research Foundation. If there are other authors, they declare that they have no known competing financial interests or personal relationships that could have appeared to influence the work reported in this paper.

## Appendix Supplementary materials

Image, application 1
