# Supplementary material for: Large-scale plasma proteomics improves prediction of heart failure among MASLD individuals: A prospective cohort study
Source: Am J Prev Cardiol. 2026 May 5;28:101662. doi: 10.1016/j.ajpc.2026.101662 (PMC13326080; doi:10.1016/j.ajpc.2026.101662)
Supplement: Supplementary file 1 [file mmc1.docx]

**Large-Scale Plasma Proteomics Improves Prediction of Heart Failure in Individuals with MASLD: A Prospective Cohort Study**

**Supplementary Figures**

**Supplementary Figure 1 —** UK Biobank MASLD-HF selection flowchart

**Supplementary Figure 2 —** LASSO selection of MASLD-HF core proteins

**Supplementary Figure 3 —** Predictive performance and cumulative incidence of heart failure evaluated under a competing risk framework

**Supplementary Tables**

**Supplementary Table 1** — Proportion of missing covariates

**Supplementary Table 2** — Baseline characteristics of study population in training set and validation set

**Supplementary Table 3** — LASSO-selected proteins associated with HF in training set

**Supplementary Table 4** — Proteins (HRs and 95% CIs) associated with HF in participants with MASLD in sensitivity analyses

**Supplementary Table 5**— Baseline characteristics of the participants in MRI-PDFF subgroup

**Supplementary Table 6** —Model predictive value in MRI-PDFF subgroup

**Supplementary Table 7**— Incremental Prognostic Value of Tiered Protein Subsets

**Supplementary Table 8** — Enrichment analysis for LASSO-selected proteins


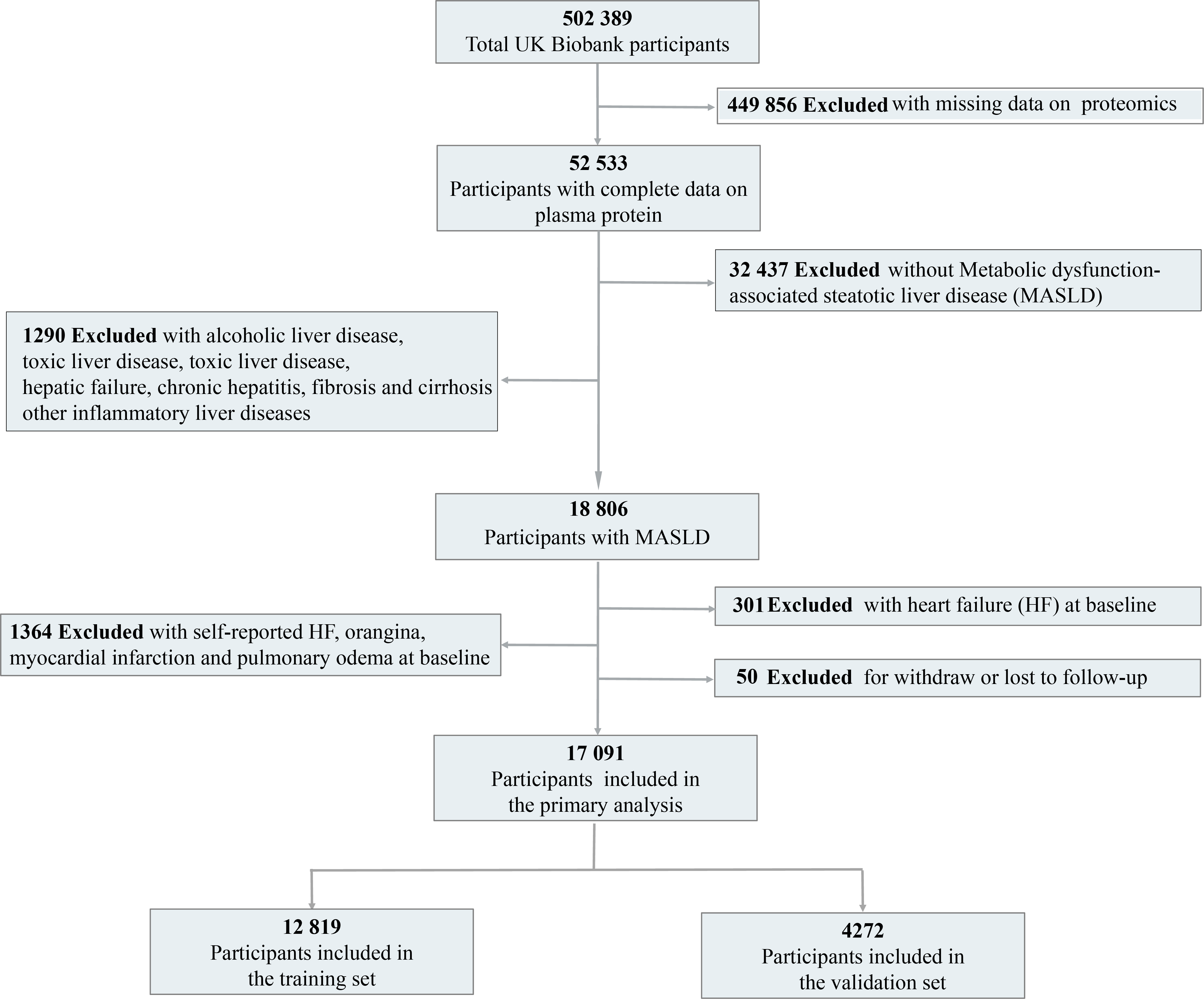


**Supplementary Figure 1 UK Biobank MASLD-HF selection flowchart**


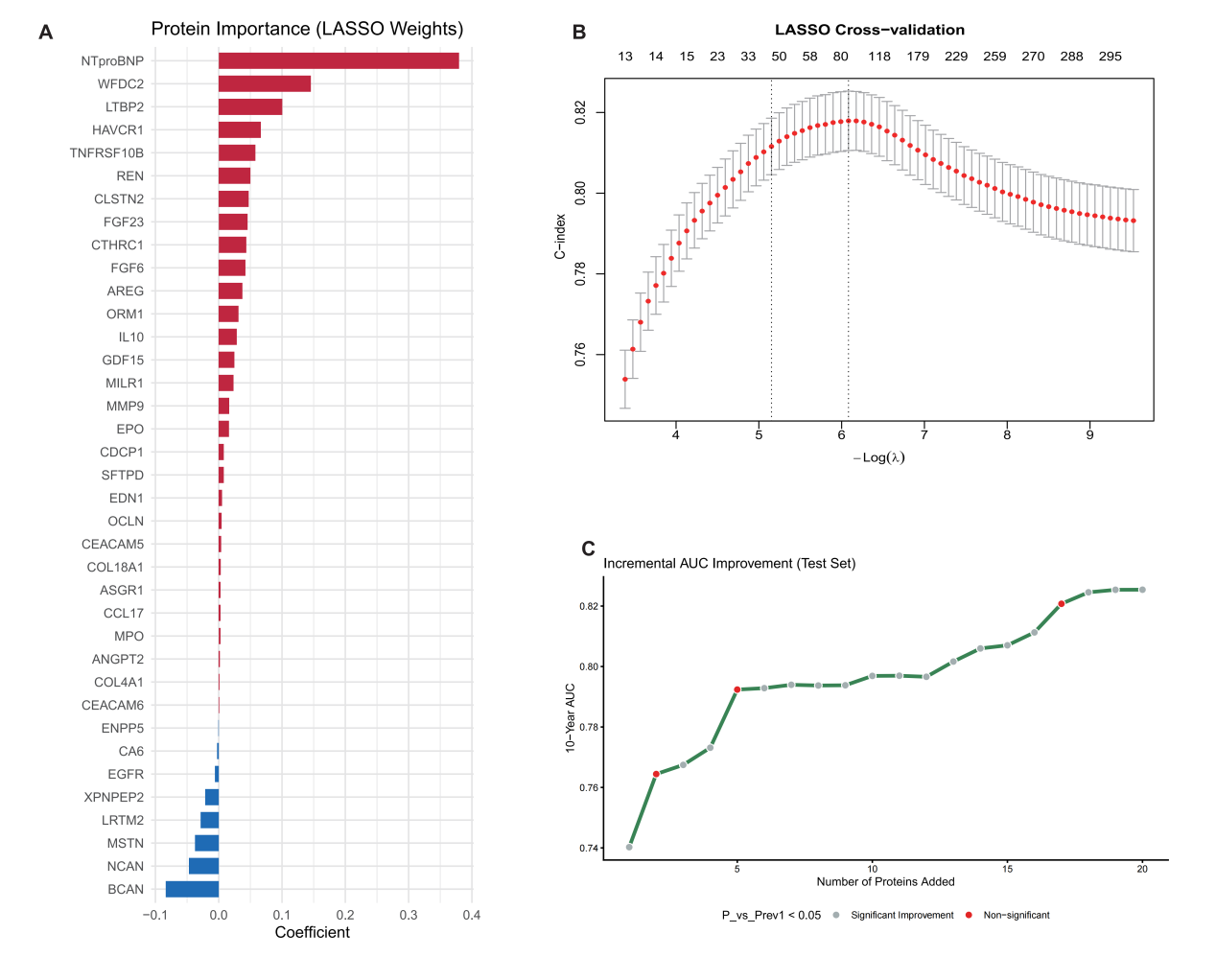


**Supplementary Figure 2 LASSO selects MASLD-HF core proteins**

(A) LASSO Cox coefficients of selected proteins. Red and blue bars indicate risk (positive) and protective (negative) factors, respectively.

(B) Ten-fold cross-validation for tuning the penalty parameter lambda. Vertical dashed lines mark lambda_1se (left, the most parsimonious model) and lambda_min (right, maximum C-index).

(C) Incremental 10-year AUC in the validation set. Proteins were sequentially added to the baseline clinical model by LASSO importance. Grey dots denote significant AUC improvement versus the preceding step (*P* < 0.05, DeLong test), whereas red dots indicate non-significance.

**
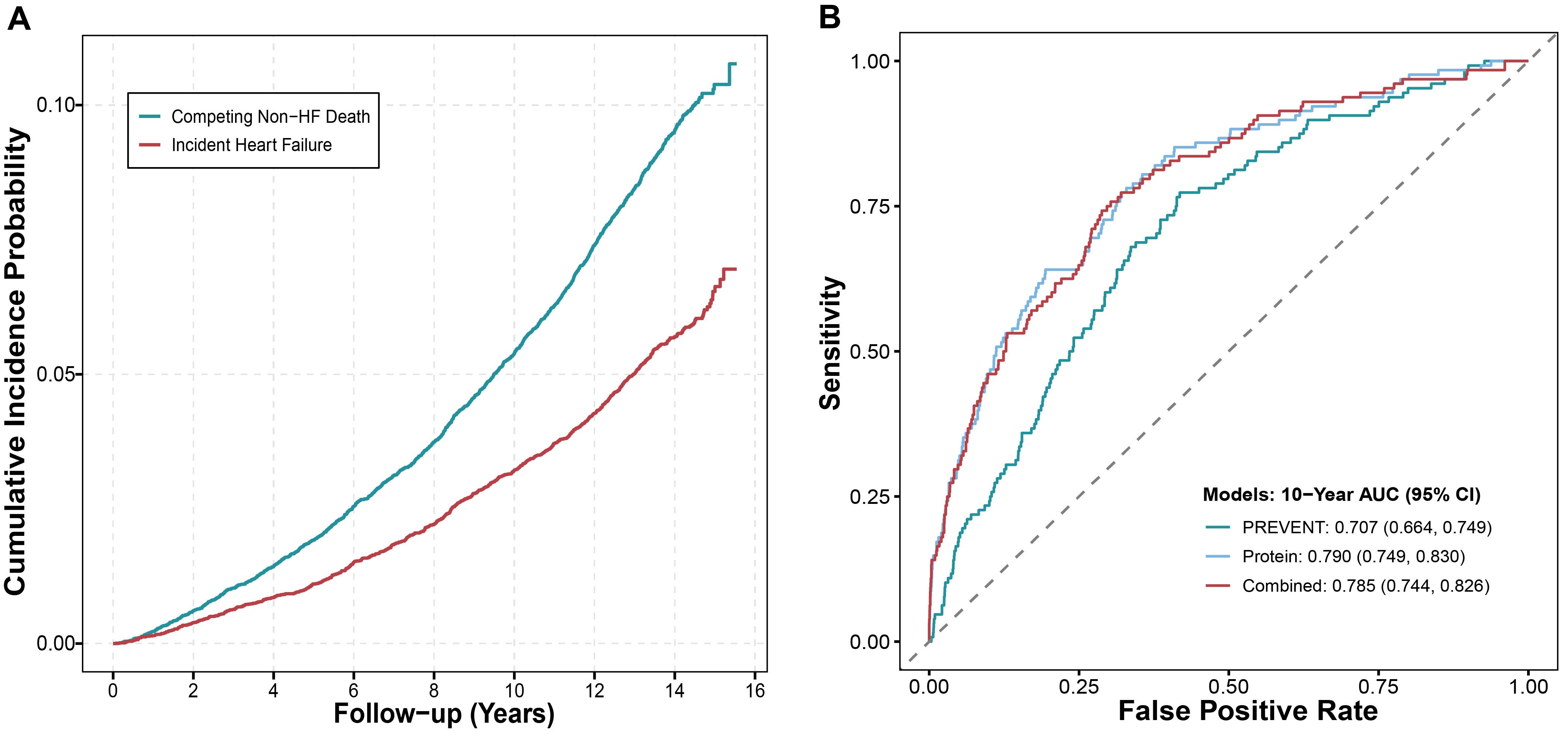
**

**Supplementary Figure 3 Predictive performance and cumulative incidence of heart failure accounting for competing risks**

(A) Competing-risk adjusted time-dependent ROC curves at 10 years for the baseline PREVENT model, the protein-only signature, and the combined model. The AUC and 95%CIs were computed using Inverse Probability of Censoring Weighting (IPCW). (B) Cumulative Incidence Function (CIF) curves illustrating the absolute probability of incident heart failure (red line, primary event) versus the competing risk of non-HF death (teal line, competing event) over the follow-up period. Abbreviations: HF, heart failure; ROC, Receiver Operating CharacteristicAUC, area under the curve; CI, confidence interval; PREVENT, predicting risk of cardiovascular disease events.

**Supplementary Table 1 Proportion of missing covariates**

| **Variable** | **Count** | **Percent (%)** |
| --- | --- | --- |
| Age | 0 | 0 |
| Sex | 0 | 0 |
| TDI | 65 | 0.12 |
| Waist | 122 | 0.23 |
| Smoking status | 173 | 0.33 |
| Ethic | 182 | 0.35 |
| BMI | 243 | 0.46 |
| TC | 2555 | 4.86 |
| Creatinine | 2575 | 4.9 |
| GGT | 2576 | 4.9 |
| TG | 2593 | 4.94 |
| Diabetes | 2813 | 5.35 |
| HbA1c | 2845 | 5.42 |
| SBP | 4410 | 8.39 |
| Albumin | 6762 | 12.87 |
| UACR | 6784 | 12.91 |
| HDL | 6789 | 12.92 |
| FPG | 6814 | 12.97 |

Abbreviations: BMI, body mass index; WC, waist circumference; SBP, systolic blood pressure; TDI, Townsend deprivation index; eGFR, estimated glomerular filtration rate; UACR, urine albumin-to-creatinine ratio; GGT gamma-glutamyl transferase; HDL high-density lipoprotein; TG, triglycerides; HbA1c, glycated hemoglobin.

**Supplementary Table 2 Baseline characteristics of study population in training set and validation set**

| **Feature** | **Total participants (N = 17,091)** | **Dataset** | | ***P*** |
| --- | --- | --- | --- | --- |
|  |  | **Training set**  **(n = 12,819)** | **Test set**  **(n = 4272)** |  |
| **Sex, n (%)** |  |  |  | 0.681 |
| Female | 6,506 (38.07) | 4,868 (37.97) | 1,638 (38.34) |  |
| Male | 10,585 (61.93) | 7,951 (62.03) | 2,634 (61.66) |  |
| **Age, years** | 57.26 (7.95) | 57.27 (7.94) | 57.20 (7.97) | 0.586 |
| **Smoking status** |  |  |  | 0.625 |
| Never | 8,525 (49.88) | 6,374 (49.72) | 2,151 (50.35) |  |
| Previous | 6,668 (39.01) | 5,006 (39.05) | 1,662 (38.90) |  |
| Current | 1,898 (11.11) | 1,439 (11.23) | 459 (10.74) |  |
| **BMI, kg/m^2^** | 31.43 (4.47) | 31.38 (4.43) | 31.58 (4.57) | 0.694 |
| **SBP, mmHg** | 142.65 (17.43) | 142.57 (17.48) | 142.90 (17.27) | 0.575 |
| **Hypertension medicine** |  |  |  | 0.671 |
| No | 12,036 (70.42) | 9,039 (70.51) | 2,997 (70.15) |  |
| Yes | 5,055 (29.58) | 3,780 (29.49) | 1,275 (29.85) |  |
| **Diabetes** |  |  |  | 0.158 |
| No | 15,916 (93.13) | 11,917 (92.96) | 3,999 (93.61) |  |
| Yes | 1,175 (6.87) | 902 (7.04) | 273 (6.39) |  |
| **eGFR, mL/min/1.73 m^2^** | 93.33 (13.97) | 93.43 (13.82) | 93.01 (14.40) | 0.094 |
| **UACR, mg/g** | 23.27 (46.18) | 23.21 (45.80) | 23.45 (47.30) | 0.308 |
| **HbA1c, mmol/mol** | 37.81 (8.55) | 37.81 (8.65) | 37.79 (8.25) | 0.907 |
| **TDI** | -0.98 (3.25) | -1.00 (3.24) | -0.94 (3.27) | 0.299 |
| **Follow-up (years)** | 13.00 (2.48) | 13.00 (2.48) | 13.00 (2.48) | 0.942 |

Continuous variables are expressed as mean ± SD, and categorical variables are described as number (percentage). Abbreviations: BMI, body mass index; SBP, systolic blood pressure; eGFR, estimated glomerular filtration rate; UACR, urine albumin-to-creatinine ratio; HbA1c, glycated hemoglobin; TDI, Abbreviations: HF, heart failure; BMI, body mass index; WC, waist circumference; SBP, systolic blood pressure; TDI, Townsend deprivation index; eGFR, estimated glomerular filtration rate; UACR, urine albumin-to-creatinine ratio; GGT gamma-glutamyl transferase; HDL high-density lipoprotein; TG, triglycerides; HbA1c, glycated hemoglobin..

**Supplementary Table 3 LASSO-selected proteins associated with HF in training set (n=12,819)**

| **Variable** | **coef** | **abs_coef** |
| --- | --- | --- |
| NTproBNP | 0.379550041 | 0.379550041 |
| WFDC2 | 0.145534033 | 0.145534033 |
| LTBP2 | 0.100518192 | 0.100518192 |
| BCAN | -0.083651378 | 0.083651378 |
| HAVCR1 | 0.066440389 | 0.066440389 |
| TNFRSF10B | 0.05781859 | 0.05781859 |
| REN | 0.050022275 | 0.050022275 |
| CLSTN2 | 0.04751516 | 0.04751516 |
| NCAN | -0.046944292 | 0.046944292 |
| FGF23 | 0.045502328 | 0.045502328 |
| CTHRC1 | 0.043437093 | 0.043437093 |
| FGF6 | 0.042109601 | 0.042109601 |
| AREG | 0.037502756 | 0.037502756 |
| MSTN | -0.037447334 | 0.037447334 |
| ORM1 | 0.03114936 | 0.03114936 |
| IL10 | 0.028682778 | 0.028682778 |
| LRTM2 | -0.028493675 | 0.028493675 |
| GDF15 | 0.024731123 | 0.024731123 |
| MILR1 | 0.023426143 | 0.023426143 |
| XPNPEP2 | -0.021264853 | 0.021264853 |
| MMP9 | 0.016700348 | 0.016700348 |
| EPO | 0.016290237 | 0.016290237 |
| CDCP1 | 0.008063605 | 0.008063605 |
| SFTPD | 0.007963942 | 0.007963942 |
| EGFR | -0.00581879 | 0.00581879 |
| EDN1 | 0.005203233 | 0.005203233 |
| OCLN | 0.004661909 | 0.004661909 |
| CEACAM5 | 0.003905167 | 0.003905167 |
| COL18A1 | 0.003132843 | 0.003132843 |
| ASGR1 | 0.00279365 | 0.00279365 |
| CCL17 | 0.00273577 | 0.00273577 |
| MPO | 0.002715135 | 0.002715135 |
| CA6 | -0.002566822 | 0.002566822 |
| ANGPT2 | 0.001977436 | 0.001977436 |
| COL4A1 | 0.00132964 | 0.00132964 |
| CEACAM6 | 0.000875758 | 0.000875758 |
| ENPP5 | -0.000634482 | 0.000634482 |

**Supplementary Table 4 Proteins (HRs and 95% CIs) associated with HF in participants with MASLD in sensitivity analyses**

| **Protein** | **Main_HR** | **Sens1_HR** | **Sens2_HR** |
| --- | --- | --- | --- |
| NTproBNP | 1.593 (1.521, 1.669) | 1.548 (1.474, 1.625) | 1.517 (1.429, 1.610) |
| WFDC2 | 2.461 (2.179, 2.779) | 2.410 (2.121, 2.739) | 2.757 (2.309, 3.293) |
| LTBP2 | 3.044 (2.597, 3.568) | 2.798 (2.364, 3.312) | 3.110 (2.565, 3.771) |
| BCAN | 0.605 (0.513, 0.714) | 0.607 (0.511, 0.720) | 0.629 (0.528, 0.749) |
| HAVCR1 | 1.558 (1.438, 1.688) | 1.529 (1.406, 1.663) | 1.555 (1.408, 1.717) |
| TNFRSF10B | 1.418 (1.327, 1.515) | 1.401 (1.305, 1.505) | 4.119 (3.364, 5.045) |
| REN | 1.231 (1.151, 1.317) | 1.234 (1.151, 1.324) | 1.160 (1.067, 1.260) |
| CLSTN2 | 1.748 (1.550, 1.972) | 1.711 (1.509, 1.941) | 1.939 (1.631, 2.305) |
| NCAN | 0.675 (0.583, 0.783) | 0.683 (0.586, 0.796) | 0.677 (0.581, 0.789) |
| FGF23 | 1.475 (1.361, 1.599) | 1.460 (1.342, 1.589) | 1.539 (1.345, 1.761) |
| CTHRC1 | 2.421 (1.981, 2.960) | 2.171 (1.753, 2.690) | 2.287 (1.791, 2.921) |
| FGF6 | 1.421 (1.185, 1.705) | 1.275 (1.027, 1.582) | 1.047 (0.664, 1.652) |
| AREG | 1.901 (1.699, 2.126) | 1.839 (1.634, 2.070) | 2.219 (1.909, 2.580) |
| MSTN | 0.723 (0.646, 0.810) | 0.753 (0.669, 0.847) | 0.710 (0.632, 0.797) |
| ORM1 | 1.791 (1.291, 2.484) | 1.749 (1.245, 2.455) | 1.943 (1.366, 2.762) |
| IL10 | 1.210 (1.142, 1.282) | 1.192 (1.121, 1.267) | 1.306 (1.191, 1.432) |
| LRTM2 | 0.479 (0.362, 0.634) | 0.492 (0.368, 0.657) | 0.428 (0.319, 0.575) |
| GDF15 | 2.055 (1.875, 2.253) | 1.995 (1.808, 2.203) | 2.612 (2.274, 2.999) |
| MILR1 | 1.396 (1.253, 1.554) | 1.392 (1.245, 1.556) | 1.384 (1.230, 1.557) |
| XPNPEP2 | 0.905 (0.857, 0.955) | 0.903 (0.854, 0.956) | 0.900 (0.851, 0.952) |
| MMP9 | 1.239 (1.125, 1.364) | 1.241 (1.123, 1.371) | 1.244 (1.127, 1.372) |
| EPO | 1.242 (1.162, 1.328) | 1.219 (1.137, 1.308) | 1.249 (1.142, 1.366) |
| CDCP1 | 1.457 (1.323, 1.605) | 1.413 (1.277, 1.563) | 1.531 (1.371, 1.711) |
| SFTPD | 1.316 (1.209, 1.433) | 1.290 (1.182, 1.409) | 1.301 (1.193, 1.419) |
| EGFR | 0.263 (0.183, 0.377) | 0.278 (0.191, 0.404) | 0.250 (0.171, 0.365) |
| EDN1 | 2.667 (2.148, 3.311) | 2.474 (1.970, 3.107) | 2.394 (1.842, 3.111) |
| OCLN | 1.808 (1.597, 2.046) | 1.749 (1.534, 1.995) | 2.024 (1.719, 2.384) |
| CEACAM5 | 1.368 (1.249, 1.499) | 1.337 (1.215, 1.472) | 1.353 (1.217, 1.505) |
| COL18A1 | 3.818 (2.958, 4.927) | 3.816 (2.928, 4.972) | 3.517 (2.660, 4.650) |
| ASGR1 | 2.090 (1.762, 2.479) | 2.079 (1.741, 2.483) | 2.217 (1.779, 2.763) |
| CCL17 | 1.179 (1.107, 1.255) | 1.174 (1.099, 1.253) | 1.181 (1.107, 1.261) |
| MPO | 1.301 (1.160, 1.459) | 1.282 (1.137, 1.444) | 1.290 (1.118, 1.487) |
| CA6 | 0.780 (0.711, 0.857) | 0.788 (0.715, 0.869) | 0.784 (0.713, 0.863) |
| ANGPT2 | 2.570 (2.215, 2.982) | 2.348 (2.007, 2.747) | 2.178 (1.831, 2.589) |
| COL4A1 | 2.031 (1.777, 2.321) | 1.960 (1.704, 2.253) | 1.867 (1.601, 2.177) |
| CEACAM6 | 1.551 (1.357, 1.772) | 1.544 (1.344, 1.775) | 1.598 (1.330, 1.921) |
| ENPP5 | 0.722 (0.648, 0.803) | 0.735 (0.656, 0.823) | 0.689 (0.596, 0.796) |

Models were adjusted for age (years, continues), sex (female, male), TDI (continues), BMI (kg/m^2^, continues), smoking status (never, previous, current), SBP (mmHg, continues) eGFR (mL/min/1.73 m^2^, continues), UACR (mg/g, continues), HbA1c (mmol/mol, continues), antihypertensive medication (yes, no), history of diabetes (yes, no); Sens1: excluding incident HF cases that occurred within the first 2 years of follow-up to mitigate potential reverse causality bias; Sens2: excluding participants with protein measurements outside the range of mean ± 3 standard deviations (SDs)

**Supplementary Table 5 Baseline characteristics of the participants in MRI-PDFF subgroup**

| **Characteristics** | **Overall** | | **Incident HF** | | | ***P* value** |
| --- | --- | --- | --- | --- | --- | --- |
|  | (n= 1481) | | No (n= 1453) | | Yes (n= 29) |  |
| **Age** | 55.13 (7.47) | | 55.06 (7.46) | | 58.90 (7.10) | 0.007 |
| **Sex** |  | |  | |  | 0.212 |
| Female | 436 (29.42) | | 431 (29.66) | | 5 (17.24) |  |
| Male | 1,046 (70.58) | | 1,022 (70.34) | | 24 (82.76) |  |
| **Smoking status** |  | |  | |  | 0.177 |
| Never | 778 (52.50) | | 764 (52.58) | | 14 (48.28) |  |
| Previous | 589 (39.74) | | 574 (39.50) | | 15 (51.72) |  |
| Current | 115 (7.76) | | 115 (7.91) | | 0 (0.00) |  |
| **TDI** | -1.67 (2.87) | | -1.67 (2.88) | | -1.75 (2.60) | 0.871 |
| **BMI, kg/m^2^** | 30.76 (3.85) | | 30.75 (3.85) | | 31.12 (4.02) | 0.634 |
| **WC, cm** | 100.87 (8.64) | | 100.82 (8.60) | | 103.55 (9.99) | 0.154 |
| **SBP, mmHg** | 140.33 (16.64) | | 140.35 (16.65) | | 139.05 (16.23) | 0.672 |
| **eGFR, mL/min/1.73m^2^** | 94.76 (12.41) | | 94.83 (12.34) | | 91.34 (15.52) | 0.239 |
| **UACR, mg/g** | 18.96 (37.23) | | 18.84 (37.25) | | 24.82 (36.55) | 0.391 |
| **GGT, U/L** | 51.37 (52.13) | | 51.46 (52.44) | | 46.80 (33.22) | 0.467 |
| **HDL, mmol/L** | 1.26 (0.29) | | 1.26 (0.29) | | 1.16 (0.21) | 0.011 |
| **TG, mg/dL** | 2.37 (1.14) | | 2.37 (1.14) | | 2.58 (1.12) | 0.318 |
| **HbA1c, mmol/mol** | 36.15 (6.07) | | 36.13 (6.01) | | 36.90 (8.73) | 0.639 |
| **Antihypertensive medication** | |  | |  | | <0.001 |
| No | 1,156 (78.00) | | 1,142 (78.60) | | 14 (48.28) |  |
| Yes | 326 (22.00) | | 311 (21.40) | | 15 (51.72) |  |
| **Diabetes** |  | |  | |  | <0.001 |
| No | 1,428 (96.36) | | 1,401 (96.42) | | 27 (93.10) |  |
| Yes | 54 (3.64) | | 52 (3.58) | | 2 (6.90) |  |

Continuous variables are expressed as mean (SD), and categorical variables are described as number (percentage). Abbreviations: HF, heart failure; BMI, body mass index; WC, waist circumference; SBP, systolic blood pressure; TDI, townsend index; eGFR, estimated glomerular filtration rate; UACR, urine albumin-to-creatinine ratio; GGT gamma-glutamyl transferase; HDL high-density lipoprotein; TG, triglycerides; HbA1c, glycated hemoglobin.

**Supplementary Table 6 Model predictive value in MRI-PDFF subgroup**

| **Model** | **PREVENT** | **Protein panel** | **Combined** |
| --- | --- | --- | --- |
| C-index (95%CI) | 0.818 (0.660–0.977) | 0.968 (0.931–1.000) | 0.976 (0.940–1.000) |
| C-index increment vs Clinical (95%CI) | Reference | 0.150 (0.120–0.180) | 0.157 (0.128–0.187) |
| Full-follow-up AUC (95%CI) | 0.820 (0.663–0.977) | 0.970 (0.931–1.000) | 0.979 (0.945–1.000) |
| AUC increment vs Clinical (95%CI) | Reference | 0.154 (0.047–0.308) | 0.161 (0.046–0.308) |
| Continuous NRI (95%CI) | Reference | -0.208 (-0.517–0.000) | 0.902 (-0.444–1.000) |
| IDI (95%CI) | Reference | 0.384 (-0.722–0.694) | 0.414 (-0.031–0.817) |

Variables in the PREVENT model including age (years, continues), sex (female, male), TDI (continues), BMI (kg/m^2^, continues), smoking status (never, previous, current), SBP (mmHg, continues) eGFR (mL/min/1.73 m^2^, continues), UACR (mg/g, continues), HbA1c (mmol/mol, continues), antihypertensive medication (yes, no), history of diabetes (yes, no). The Protein panel was only developed by integrating LASSO-selected proteins, while the Protein+PREVENT model incorporated LASSO-selected proteins alongside the PREVENT variables. Abbreviations: PREVENT: predicting risk of cardiovascular disease events; AUC, area under curve; NRI, net reclassification improvement; IDI, integrated discrimination improvement

.

**Supplementary Table 7 Incremental Prognostic Value of Tiered Protein Subsets**

| **Model** | **C-index (95%CI)** | **ΔC-index (95%CI)** |
| --- | --- | --- |
| PREVENT | 0.723 (0.691–0.755) | — |
| Only NT-proBNP | 0.674 (0.635–0.714) | -0.049 (-0.068 to -0.029) |
| Top5 Protein | 0.769 (0.737–0.801) | 0.046 (0.027–0.064) |
| Protein panel | 0.802 (0.772–0.832) | 0.078 (0.060–0.096) |
| PREVENT + NT-proBNP | 0.744 (0.709–0.778) | 0.021 (0.002–0.040) |
| PREVENT + Top5 Protein | 0.782 (0.751–0.813) | 0.059 (0.041–0.077) |
| Combined | 0.805 (0.776–0.835) | 0.082 (0.064–0.100) |

The baseline PREVENT model serves as the reference for ΔC-index. "Top5 Protein" includes NT-proBNP, WFDC2, LTBP2, BCAN, and HAVCR1, which had the highest absolute coefficients in the LASSO model. "Protein panel" refers to all 37 LASSO-selected proteins. "Combined" integrates the PREVENT model with the Protein panel. Abbreviations: CI, confidence interval; LASSO, least absolute shrinkage and selection operator; NT-proBNP, N-terminal pro-B-type natriuretic peptide; PREVENT: predicting risk of cardiovascular disease events.

**Supplementary Table 8 Enrichment analysis for LASSO-selected proteins**

| **Description** | **FoldEnrichment** | ***P* value** | **Genes in enriched term** | **Ontology** |
| --- | --- | --- | --- | --- |
| Response to glucocorticoid | 19.70 | 5.11E-06 | AREG/MSTN/IL10/EPO/EDN1 | BP |
| Response to corticosteroid | 17.01 | 1.05E-05 | AREG/MSTN/IL10/EPO/EDN1 | BP |
| Response to activity | 24.95 | 1.93E-05 | MSTN/IL10/EDN1/ANGPT2 | BP |
| Response to lipopolysaccharide | 8.98 | 4.76E-05 | REN/IL10/MMP9/EPO/EDN1/MPO | BP |
| Multi-organism reproductive process | 12.07 | 5.43E-05 | MSTN/MMP9/EPO/EDN1/ANGPT2 | BP |
| Multi-multicellular organism process | 11.54 | 6.72E-05 | MSTN/MMP9/EPO/EDN1/ANGPT2 | BP |
| Response to molecule of bacterial origin | 8.38 | 6.98E-05 | REN/IL10/MMP9/EPO/EDN1/MPO | BP |
| Response to testosterone | 36.55 | 7.48E-05 | MSTN/EPO/EDN1 | BP |
| Response to dexamethasone | 35.72 | 8.02E-05 | MSTN/EPO/EDN1 | BP |
| Positive regulation of vascular associated smooth muscle cell proliferation | 29.65 | 1.40E-04 | IL10/MMP9/EDN1 | BP |
| Response to acid chemical | 14.55 | 1.59E-04 | EGFR/EDN1/COL18A1/COL4A1 | BP |
| Regulation of excretion | 95.25 | 1.93E-04 | EDN1/NPPB | BP |
| Response to peptide hormone | 6.82 | 2.16E-04 | REN/AREG/MSTN/IL10/GDF15/EDN1 | BP |
| Positive regulation of urine volume | 87.31 | 2.31E-04 | EDN1/NPPB | BP |
| Renal sodium excretion | 87.31 | 2.31E-04 | EDN1/NPPB | BP |
| Chemotaxis | 6.63 | 2.51E-04 | MSTN/IL10/SFTPD/EDN1/CCL17/ANGPT2 | BP |
| Positive regulation of mapk cascade | 6.63 | 2.51E-04 | FGF23/FGF6/GDF15/EPO/EGFR/EDN1 | BP |
| Taxis | 6.60 | 2.56E-04 | MSTN/IL10/SFTPD/EDN1/CCL17/ANGPT2 | BP |
| Positive regulation of heterotypic cell-cell adhesion | 80.60 | 2.73E-04 | IL10/CEACAM6 | BP |
| Embryo implantation | 22.14 | 3.34E-04 | MSTN/MMP9/EPO | BP |
| Regulation of response to wounding | 11.71 | 3.65E-04 | IL10/EDN1/OCLN/ANGPT2 | BP |
| Response to uv-a | 69.85 | 3.66E-04 | MMP9/EGFR | BP |
| Cell chemotaxis | 7.94 | 3.84E-04 | MSTN/IL10/SFTPD/EDN1/CCL17 | BP |
| Renal system development | 7.84 | 4.06E-04 | REN/MMP9/EDN1/ANGPT2/COL4A1 | BP |
| Response to steroid hormone | 7.64 | 4.58E-04 | AREG/MSTN/IL10/EPO/EDN1 | BP |
| Female pregnancy | 10.64 | 5.24E-04 | MSTN/MMP9/EPO/ANGPT2 | BP |
| Morphogenesis of a branching epithelium | 10.53 | 5.44E-04 | AREG/IL10/EDN1/COL4A1 | BP |
| Regulation of mast cell activation | 55.15 | 5.94E-04 | HAVCR1/MILR1 | BP |
| Negative regulation of anoikis | 55.15 | 5.94E-04 | CEACAM5/CEACAM6 | BP |
| Renal tubular secretion | 52.39 | 6.59E-04 | EDN1/NPPB | BP |
| Morphogenesis of a branching structure | 9.79 | 7.14E-04 | AREG/IL10/EDN1/COL4A1 | BP |
| Regulation of urine volume | 49.89 | 7.27E-04 | EDN1/NPPB | BP |
| Regulation of vascular associated smooth muscle cell proliferation | 16.54 | 7.84E-04 | IL10/MMP9/EDN1 | BP |
| Response to leptin | 47.63 | 7.99E-04 | FGF23/EDN1 | BP |
| Vascular associated smooth muscle cell proliferation | 16.20 | 8.33E-04 | IL10/MMP9/EDN1 | BP |
| Positive regulation of smooth muscle cell proliferation | 16.04 | 8.58E-04 | IL10/MMP9/EDN1 | BP |
| Regulation of heterotypic cell-cell adhesion | 45.56 | 8.74E-04 | IL10/CEACAM6 | BP |
| Erbb2 signaling pathway | 45.56 | 8.74E-04 | AREG/EGFR | BP |
| Leukocyte migration | 6.60 | 8.88E-04 | MSTN/IL10/GDF15/SFTPD/EDN1 | BP |
| Response to mechanical stimulus | 9.19 | 9.05E-04 | TNFRSF10B/EDN1/MPO/ANGPT2 | BP |
| Regulation of renal system process | 43.66 | 9.52E-04 | EDN1/NPPB | BP |
| Response to salt | 43.66 | 9.52E-04 | FGF23/EDN1 | BP |
| Leukocyte chemotaxis | 8.84 | 1.04E-03 | MSTN/IL10/SFTPD/EDN1 | BP |
| Regulation of anoikis | 40.30 | 1.12E-03 | CEACAM5/CEACAM6 | BP |
| Regulation of systemic arterial blood pressure | 14.03 | 1.26E-03 | REN/EDN1/NPPB | BP |
| Response to estradiol | 13.91 | 1.29E-03 | AREG/IL10/EGFR | BP |
| Positive regulation of lamellipodium assembly | 37.42 | 1.30E-03 | MSTN/OCLN | BP |
| Muscle cell proliferation | 8.32 | 1.31E-03 | MSTN/IL10/MMP9/EDN1 | BP |
| Positive regulation of phosphorylation | 8.25 | 1.35E-03 | TNFRSF10B/AREG/MMP9/EGFR | BP |
| Negative regulation of intrinsic apoptotic signaling pathway | 13.67 | 1.36E-03 | IL10/MMP9/EPO | BP |
| Ameboidal-type cell migration | 8.19 | 1.39E-03 | MSTN/MMP9/EDN1/OCLN | BP |
| Glomerulus vasculature development | 34.93 | 1.49E-03 | EDN1/ANGPT2 | BP |
| Prostaglandin biosynthetic process | 33.80 | 1.59E-03 | CTHRC1/EDN1 | BP |
| Excretion | 33.80 | 1.59E-03 | EDN1/NPPB | BP |
| Renal system vasculature development | 32.74 | 1.69E-03 | EDN1/ANGPT2 | BP |
| Kidney vasculature development | 32.74 | 1.69E-03 | EDN1/ANGPT2 | BP |
| Prostanoid biosynthetic process | 31.75 | 1.80E-03 | CTHRC1/EDN1 | BP |
| Epidermal growth factor receptor signaling pathway | 12.38 | 1.81E-03 | AREG/MMP9/EGFR | BP |
| Cell-cell adhesion via plasma-membrane adhesion molecules | 7.51 | 1.90E-03 | CLSTN2/IL10/CEACAM5/CEACAM6 | BP |
| Response to amino acid | 12.09 | 1.93E-03 | EGFR/EDN1/COL4A1 | BP |
| Vascular process in circulatory system | 7.40 | 2.00E-03 | REN/EDN1/OCLN/NPPB | BP |
| Anoikis | 29.10 | 2.14E-03 | CEACAM5/CEACAM6 | BP |
| Cellular response to estradiol stimulus | 29.10 | 2.14E-03 | IL10/EGFR | BP |
| Positive regulation of lamellipodium organization | 29.10 | 2.14E-03 | MSTN/OCLN | BP |
| Regulation of wound healing | 11.31 | 2.34E-03 | EDN1/OCLN/ANGPT2 | BP |
| Extracellular matrix | 9.78 | 2.27E-07 | LTBP2/BCAN/NCAN/CTHRC1/GDF15/MMP9/COL18A1/ANGPT2/COL4A1 | CC |
| External encapsulating structure | 9.76 | 2.31E-07 | LTBP2/BCAN/NCAN/CTHRC1/GDF15/MMP9/COL18A1/ANGPT2/COL4A1 | CC |
| Collagen trimer | 25.07 | 1.91E-05 | CTHRC1/SFTPD/COL18A1/COL4A1 | CC |
| Perineuronal net | 107.81 | 1.49E-04 | BCAN/NCAN | CC |
| Perisynaptic extracellular matrix | 98.00 | 1.82E-04 | BCAN/NCAN | CC |
| Synapse-associated extracellular matrix | 89.84 | 2.18E-04 | BCAN/NCAN | CC |
| Endocytic vesicle | 7.47 | 5.12E-04 | AREG/SFTPD/EGFR/OCLN/MPO | CC |
| Clathrin-coated endocytic vesicle | 17.20 | 7.01E-04 | AREG/SFTPD/EGFR | CC |
| Clathrin-coated vesicle | 9.67 | 7.52E-04 | AREG/SFTPD/EGFR/EDN1 | CC |
| Specialized extracellular matrix | 49.00 | 7.56E-04 | BCAN/NCAN | CC |
| Golgi lumen | 14.84 | 1.08E-03 | BCAN/NCAN/FGF23 | CC |
| Endoplasmic reticulum lumen | 6.76 | 2.80E-03 | FGF23/EDN1/COL18A1/COL4A1 | CC |
| Coated vesicle | 6.68 | 2.93E-03 | AREG/SFTPD/EGFR/EDN1 | CC |
| Vacuolar lumen | 9.03 | 4.41E-03 | BCAN/NCAN/MPO | CC |
| Tertiary granule lumen | 19.60 | 4.67E-03 | ORM1/MMP9 | CC |
| Multivesicular body | 15.85 | 7.06E-03 | SFTPD/EGFR | CC |
| Clathrin-coated endocytic vesicle membrane | 14.18 | 8.76E-03 | AREG/EGFR | CC |
| Cytokine activity | 15.25 | 3.06E-07 | AREG/MSTN/IL10/GDF15/EPO/EDN1/CCL17 | MF |
| Growth factor activity | 19.20 | 6.12E-07 | FGF23/FGF6/AREG/MSTN/IL10/GDF15 | MF |
| Glycosaminoglycan binding | 10.80 | 9.19E-05 | LTBP2/BCAN/NCAN/MSTN/MPO | MF |
| Hormone activity | 15.59 | 1.22E-04 | GDF15/EPO/EDN1/NPPB | MF |
| Growth factor receptor binding | 14.81 | 1.48E-04 | FGF23/FGF6/AREG/IL10 | MF |
| Protein kinase activator activity | 13.82 | 1.93E-04 | AREG/MSTN/EPO/EGFR | MF |
| Kinase activator activity | 12.96 | 2.48E-04 | AREG/MSTN/EPO/EGFR | MF |
| Extracellular matrix structural constituent | 11.85 | 3.48E-04 | LTBP2/CTHRC1/COL18A1/COL4A1 | MF |
| Hyaluronic acid binding | 43.20 | 9.72E-04 | BCAN/NCAN | MF |
| Fibroblast growth factor receptor binding | 38.40 | 1.23E-03 | FGF23/FGF6 | MF |
| Protein kinase regulator activity | 7.57 | 1.85E-03 | AREG/MSTN/EPO/EGFR | MF |
| Carbohydrate binding | 7.35 | 2.05E-03 | BCAN/NCAN/SFTPD/ASGR1 | MF |
| Growth factor binding | 11.44 | 2.27E-03 | LTBP2/EGFR/COL4A1 | MF |
| Protein tyrosine kinase activator activity | 27.28 | 2.43E-03 | AREG/EGFR | MF |
| Kinase regulator activity | 6.73 | 2.83E-03 | AREG/MSTN/EPO/EGFR | MF |
| Extracellular matrix structural constituent conferring tensile strength | 24.69 | 2.97E-03 | COL18A1/COL4A1 | MF |
| Heparin binding | 9.15 | 4.25E-03 | LTBP2/MSTN/MPO | MF |
| Extracellular matrix organization | 9.48 | 2.80E-05 | LTBP2/BCAN/NCAN/MMP9/COL4A1/CEACAM6 | Reactome |
| Signaling by activated point mutants of fgfr1 | 88.46 | 2.18E-04 | FGF23/FGF6 | Reactome |
| Fgfr1c ligand binding and activation | 73.72 | 3.18E-04 | FGF23/FGF6 | Reactome |
| Fgfr2c ligand binding and activation | 73.72 | 3.18E-04 | FGF23/FGF6 | Reactome |
| Ds-gag biosynthesis | 73.72 | 3.18E-04 | BCAN/NCAN | Reactome |
| Ecm proteoglycans | 20.73 | 3.84E-04 | BCAN/NCAN/COL4A1 | Reactome |
| Fgfr4 ligand binding and activation | 63.19 | 4.38E-04 | FGF23/FGF6 | Reactome |
| Constitutive signaling by aberrant pi3k in cancer | 19.80 | 4.40E-04 | FGF23/FGF6/AREG | Reactome |
| Phospholipase c-mediated cascade: Fgfr1 | 58.97 | 5.04E-04 | FGF23/FGF6 | Reactome |
| Phospholipase c-mediated cascade; fgfr4 | 58.97 | 5.04E-04 | FGF23/FGF6 | Reactome |
| Fibronectin matrix formation | 55.29 | 5.76E-04 | COL4A1/CEACAM6 | Reactome |
| Fgfr1 ligand binding and activation | 55.29 | 5.76E-04 | FGF23/FGF6 | Reactome |
| Activated point mutants of fgfr2 | 55.29 | 5.76E-04 | FGF23/FGF6 | Reactome |
| Phospholipase c-mediated cascade; fgfr2 | 52.04 | 6.51E-04 | FGF23/FGF6 | Reactome |
| Pi-3k cascade:Fgfr1 | 52.04 | 6.51E-04 | FGF23/FGF6 | Reactome |
| Pi-3k cascade:Fgfr4 | 52.04 | 6.51E-04 | FGF23/FGF6 | Reactome |
| Defective b4galt7 causes eds, progeroid type | 49.14 | 7.32E-04 | BCAN/NCAN | Reactome |
| Defective b3gat3 causes jdssdhd | 49.14 | 7.32E-04 | BCAN/NCAN | Reactome |
| Defective b3galt6 causes edsp2 and semdjl1 | 49.14 | 7.32E-04 | BCAN/NCAN | Reactome |
| Fgfr2 ligand binding and activation | 46.56 | 8.17E-04 | FGF23/FGF6 | Reactome |
| Shc-mediated cascade:Fgfr1 | 46.56 | 8.17E-04 | FGF23/FGF6 | Reactome |
| Pi-3k cascade:Fgfr2 | 46.56 | 8.17E-04 | FGF23/FGF6 | Reactome |
| Shc-mediated cascade:Fgfr4 | 46.56 | 8.17E-04 | FGF23/FGF6 | Reactome |
| Pi5p, pp2a and ier3 regulate pi3k/akt signaling | 15.61 | 8.84E-04 | FGF23/FGF6/AREG | Reactome |
| Cs-gag biosynthesis | 44.23 | 9.06E-04 | BCAN/NCAN | Reactome |
| Cs/ds degradation | 44.23 | 9.06E-04 | BCAN/NCAN | Reactome |
| Pi3k/akt signaling in cancer | 15.43 | 9.14E-04 | FGF23/FGF6/AREG | Reactome |
| Frs-mediated fgfr1 signaling | 42.12 | 1.00E-03 | FGF23/FGF6 | Reactome |
| Shc-mediated cascade:Fgfr2 | 42.12 | 1.00E-03 | FGF23/FGF6 | Reactome |
| Frs-mediated fgfr4 signaling | 42.12 | 1.00E-03 | FGF23/FGF6 | Reactome |
| Negative regulation of the pi3k/akt network | 14.58 | 1.08E-03 | FGF23/FGF6/AREG | Reactome |
| Fgfr1 mutant receptor activation | 40.21 | 1.10E-03 | FGF23/FGF6 | Reactome |
| Frs-mediated fgfr2 signaling | 38.46 | 1.20E-03 | FGF23/FGF6 | Reactome |
| Downstream signaling of activated fgfr4 | 38.46 | 1.20E-03 | FGF23/FGF6 | Reactome |
| Downstream signaling of activated fgfr2 | 35.38 | 1.42E-03 | FGF23/FGF6 | Reactome |
| Downstream signaling of activated fgfr1 | 34.02 | 1.54E-03 | FGF23/FGF6 | Reactome |
| Glycosaminoglycan-protein linkage region biosynthesis | 32.76 | 1.66E-03 | BCAN/NCAN | Reactome |
| Negative regulation of fgfr4 signaling | 32.76 | 1.66E-03 | FGF23/FGF6 | Reactome |
| Negative regulation of fgfr1 signaling | 31.59 | 1.78E-03 | FGF23/FGF6 | Reactome |
| Signaling by fgfr1 in disease | 31.59 | 1.78E-03 | FGF23/FGF6 | Reactome |
| Negative regulation of fgfr2 signaling | 30.50 | 1.91E-03 | FGF23/FGF6 | Reactome |
| Fgfr2 mutant receptor activation | 27.64 | 2.33E-03 | FGF23/FGF6 | Reactome |
| Signaling by receptor tyrosine kinases | 5.11 | 2.43E-03 | FGF23/FGF6/AREG/MMP9/COL4A1 | Reactome |
| Ncam1 interactions | 26.02 | 2.63E-03 | NCAN/COL4A1 | Reactome |
| Signaling by fgfr4 | 26.02 | 2.63E-03 | FGF23/FGF6 | Reactome |
| Degradation of the extracellular matrix | 10.37 | 2.87E-03 | BCAN/MMP9/COL4A1 | Reactome |
| Chondroitin sulfate/dermatan sulfate metabolism | 24.57 | 2.94E-03 | BCAN/NCAN | Reactome |
| Pi3k cascade | 23.91 | 3.10E-03 | FGF23/FGF6 | Reactome |
| Diseases associated with glycosaminoglycan metabolism | 22.68 | 3.44E-03 | BCAN/NCAN | Reactome |
| Signaling by fgfr2 in disease | 22.12 | 3.62E-03 | FGF23/FGF6 | Reactome |
| Irs-mediated signalling | 21.58 | 3.80E-03 | FGF23/FGF6 | Reactome |
| Insulin receptor signalling cascade | 21.06 | 3.98E-03 | FGF23/FGF6 | Reactome |
| Signaling by fgfr1 | 20.57 | 4.17E-03 | FGF23/FGF6 | Reactome |
| Igf1r signaling cascade | 19.66 | 4.56E-03 | FGF23/FGF6 | Reactome |
| Irs-related events triggered by igf1r | 19.66 | 4.56E-03 | FGF23/FGF6 | Reactome |
| Signaling by type 1 insulin-like growth factor 1 receptor (igf1r) | 19.23 | 4.76E-03 | FGF23/FGF6 | Reactome |
| Ncam signaling for neurite out-growth | 17.35 | 5.83E-03 | NCAN/COL4A1 | Reactome |
| Signaling by fgfr in disease | 17.01 | 6.05E-03 | FGF23/FGF6 | Reactome |
| Assembly of collagen fibrils and other multimeric structures | 16.69 | 6.28E-03 | MMP9/COL4A1 | Reactome |
| Extra-nuclear estrogen signaling | 15.25 | 7.48E-03 | AREG/MMP9 | Reactome |
| Diseases of signal transduction by growth factor receptors and second messengers | 5.00 | 7.56E-03 | FGF23/FGF6/AREG/IL10 | Reactome |
| Collagen degradation | 14.99 | 7.74E-03 | MMP9/COL4A1 | Reactome |
| Signaling by fgfr2 | 14.74 | 7.99E-03 | FGF23/FGF6 | Reactome |
| Pip3 activates akt signaling | 6.63 | 9.92E-03 | FGF23/FGF6/AREG | Reactome |
| Signaling by insulin receptor | 13.01 | 1.02E-02 | FGF23/FGF6 | Reactome |
| Signaling by fgfr | 11.95 | 1.20E-02 | FGF23/FGF6 | Reactome |
| Raf/map kinase cascade | 5.92 | 1.35E-02 | FGF23/FGF6/AREG | Reactome |
| Collagen formation | 11.06 | 1.39E-02 | MMP9/COL4A1 | Reactome |
| Mapk1/mapk3 signaling | 5.85 | 1.40E-02 | FGF23/FGF6/AREG | Reactome |
| Neutrophil degranulation | 4.11 | 1.47E-02 | ORM1/MMP9/MPO/CEACAM6 | Reactome |
| Intracellular signaling by second messengers | 5.62 | 1.55E-02 | FGF23/FGF6/AREG | Reactome |
| Post-translational modification: Synthesis of gpi-anchored proteins | 10.17 | 1.63E-02 | XPNPEP2/CEACAM5 | Reactome |
| Interleukin-4 and interleukin-13 signaling | 10.17 | 1.63E-02 | IL10/MMP9 | Reactome |
| Mapk family signaling cascades | 5.16 | 1.94E-02 | FGF23/FGF6/AREG | Reactome |
| Pi3k-akt signaling pathway | 6.91 | 4.17E-05 | FGF23/FGF6/AREG/EPO/EGFR/ANGPT2/COL4A1 | KEGG |
| Cytokine-cytokine receptor interaction | 7.11 | 1.42E-04 | TNFRSF10B/MSTN/IL10/GDF15/EPO/CCL17 | KEGG |
| Hif-1 signaling pathway | 12.83 | 2.35E-04 | EPO/EGFR/EDN1/ANGPT2 | KEGG |
| Relaxin signaling pathway | 10.84 | 4.47E-04 | MMP9/EGFR/EDN1/COL4A1 | KEGG |
| Melanoma | 14.56 | 1.08E-03 | FGF23/FGF6/EGFR | KEGG |
| Mapk signaling pathway | 5.79 | 1.42E-03 | FGF23/FGF6/AREG/EGFR/ANGPT2 | KEGG |
| Rap1 signaling pathway | 6.66 | 2.74E-03 | FGF23/FGF6/EGFR/ANGPT2 | KEGG |
| Viral protein interaction with cytokine and cytokine receptor | 10.49 | 2.77E-03 | TNFRSF10B/IL10/CCL17 | KEGG |
| Protein digestion and absorption | 10.18 | 3.02E-03 | XPNPEP2/COL18A1/COL4A1 | KEGG |
| Ras signaling pathway | 5.92 | 4.17E-03 | FGF23/FGF6/EGFR/ANGPT2 | KEGG |

Terms were considered significant after Bonferroni correction for the total number of pathways tested (N = 184), *P* < 0.05/184.
